# The importance of prosthetic planning for implant-supported dentures in esthetic zones – A case report

**DOI:** 10.1016/j.ijscr.2018.11.059

**Published:** 2018-11-27

**Authors:** Gustavo Vargas Da Silva Salomão, Fernando Toledo Santos, Sergio Allegrini Junior

**Affiliations:** School of Dentistry, Ibirapuera University (Unib), Av. Interlagos, 1329, Chácara Flora, São Paulo, SP 04661-100, Brazil

**Keywords:** Dental implants, Cement-retained prosthesis, Dental esthetics, Implant-supported denture, Case report

## Abstract

**Introduction:**

Implant-supported dentures in esthetic zones pose a great challenge to dental surgeons, since important factors are involved in treatment success, such as positioning of the implants, morphology of soft tissues, dental esthetics, and biomechanics of the prosthetic structure.

**Presentation of case:**

This clinical report describes the case of a patient previously subjected to dental surgery for implant placement on tooth #11 and subsequent prosthetic crown placement. The patient reported poor esthetics due to gingival retraction and changes on the surface of the ceramic material. After proper multidisciplinary planning, the prosthesis was remanufactured, with the selection of new components and periodontal surgery using a modified technique for the handling of soft tissues. After nine postoperative months, the patient had a better gingival alignment and reported esthetic and functional satisfaction.

**Discussion:**

After assessing the positioning of implants, it is important to select prosthetic components that are suitable to each clinical case, as they will guarantee clinical success for long time periods. In order to obtain a better gingival contour and avoid an extensive surgical procedure, two techniques were used: the pinhole surgical technique, in which the gingiva is repositioned without the use of grafts; and Tarnow’s technique, in which a semilunar incision is made in soft tissues.

**Conclusion:**

The multidisciplinary approach, combining prosthetic and periodontal procedures, proved to be efficient, improving esthetic outcomes.

## Highlights

• Prosthetic planning prior to surgical treatment is crucial for the success of implant-supported prostheses.
• Poor rehabilitation planning can lead to gingival retraction and fracture of prosthetic components.
• A multidisciplinary approach is of utmost importance to achieve the desired results.

## 1 Introduction

Nowadays, satisfactory esthetic outcomes have become a burgeoning demand at dental offices. Owing to the major breakthroughs in techniques and materials at hand, patients have been overly demanding in terms of esthetics, presenting dental surgeons with a great challenge.

When dental implants are placed in esthetic zones, some important determining factors for a successful outcome should be taken into account, such as positioning of implants, positioning of soft tissues, dental esthetics, and biomechanics of the dental prosthesis [1,2].

Prosthetic planning prior to implant placement is of utmost importance as it will determine the position in which the implant has to be placed. If only the amount of bone tissue in the recipient zone is taken into consideration, without prosthetic planning, the implant could be mispositioned and the prosthesis misangulated, compromising esthetics and the function of the final prosthetic crown [1].

The characteristics of soft tissues around the implants are crucial for oral rehabilitations and patient satisfaction [2]. Inflamed tissues, due to large plaque accumulation, to inappropriate selection of prosthetic components, or to any residue from the material (cement for crowns), may lead to gingival conditions, such as retraction of peri-implant tissues or development of peri-implantitis [[3], [4], [5]].

Success is closely related to the esthetics of rehabilitations [6]. Sometimes, it is necessary to perform periodontal surgeries before, during, or after implant placement so that the position of gingival tissue will be properly esthetic and functional.

The functional behavior of implant-supported dentures plays an extremely important role in that any change could lead to treatment failure. To prevent these changes, the following should be taken into consideration: prosthetic components, the cement-retained model (with better esthetic outcomes because there is no screw access hole) [1,[3], [4], [5],^7^] or screw-retained model (in which maintenance is easier because of access to the screw) [8], occlusal adjustment, and biomechanical factors [1]. This case report from our institute demonstrated the need for prosthetic planning prior to surgery and the importance of a multidisciplinary approach.

## 2 Presentation of the clinical case

**Fig. 1:**
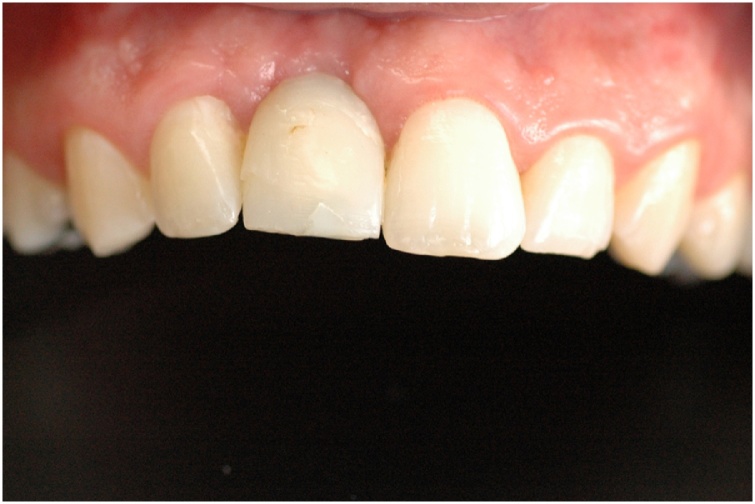
Initial clinical aspect.

**Fig. 2:**
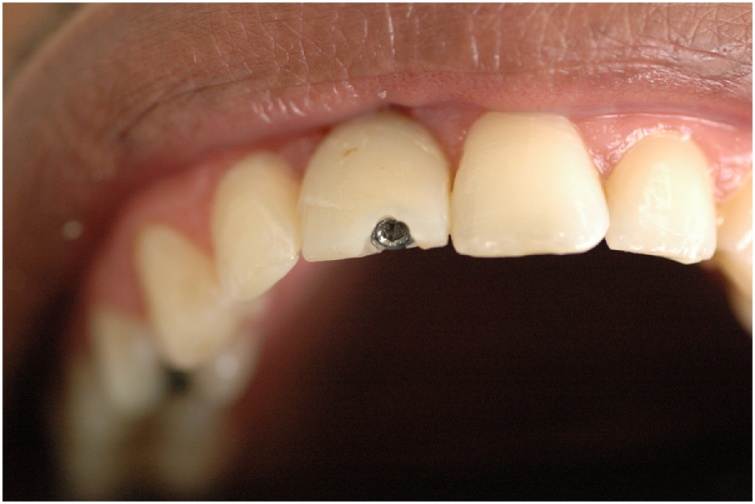
Screw access hole through the buccal surface.

**Fig. 3:**
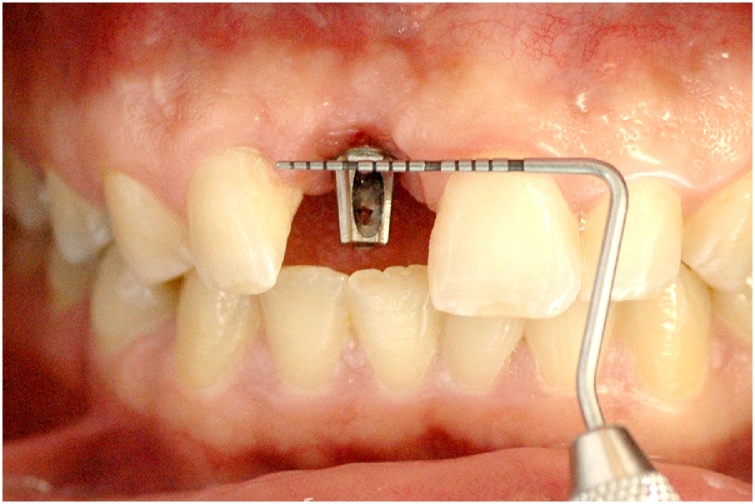
Assessment of gingival retraction in the buccal region, compared with the cervical region of tooth #21.

This manuscript was constructed in compliance with consensus-based surgical case report guidelines (SCARE) [9]. W. S. S. P., 29-year-old male patient, nonsmoker, nondrinker, not taking any medication, and with no relevant family history, with an implant-supported crown in central incisor #11 fabricated 8 months before, was dissatisfied with the final esthetic outcome (Fig. 1). In his dental history, the patient reported having undergone the surgical procedure for placement of osseointegrated implant. The prosthetic crown was placed 6 months after healing. Gingival retraction was observed after 8 months. Clinical examination revealed a screw-retained prosthesis through the incisal surface (Fig. 2), covered with photoactivated composite resin, stained over time by coloring agents. The gingiva was vertically retracted in the buccal region of the crown (approximately 2 mm), when compared with the cervical region of tooth #21 (Fig. 3). Ceramic coating of the prosthetic element revealed cracks on its buccal surface, whereas on the palatal surface there was excessive wear with prosthetic exposure of the metal coping. After removal of the implant-supported crown, no abutment was observed, which was considered to be the probable cause for treatment failure.

Plaster study models were mounted in a semi-adjustable articulator and the periodontal surgical procedure was then planned for repositioning and leveling of the gingiva to its buccal region. As to the manufacture of the prosthesis, due to the position of the previously placed endosseous implant, the final prosthetic rehabilitation should be targeted at a cement-retained prosthesis with selection of an intermediate abutment at an angle of 15°, a 0.8 mm band, and height of 6 mm. The laboratory technician was asked to manufacture a temporary acrylic resin crown in order to provide an ideal emergence profile for the implant-supported crown, helping with gingival healing after periodontal surgery.

**Fig. 4:**
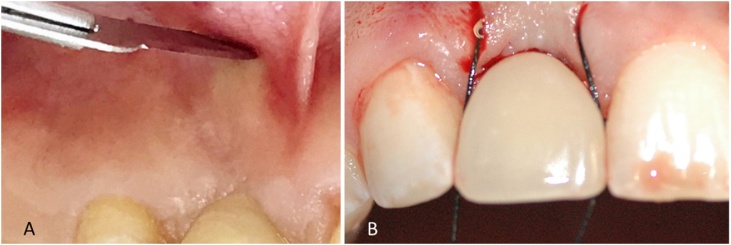
4A – Horizontal incision in the alveolar mucosa; 4B –Gingival repositioning with combination of Tarnow’s and pinhole surgical techniques.

**Fig. 5:**
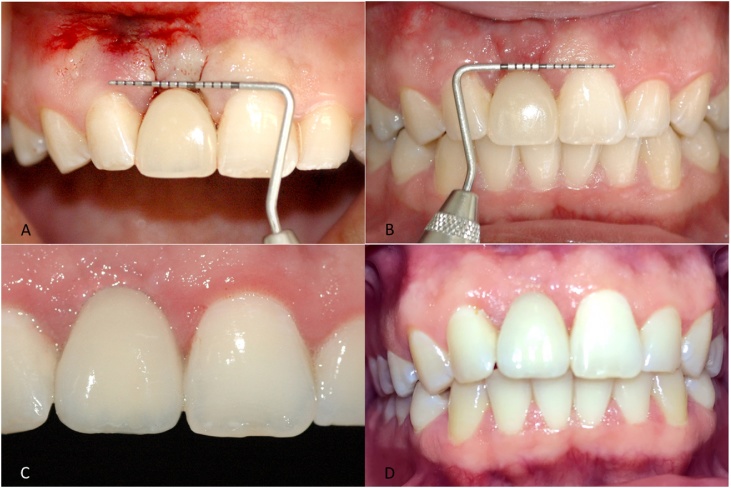
5A – Confirmation of gingival position after periodontal surgery. The papillae were preserved; 5B –10 days after surgery showing healthy gingival tissue without gingival recession; 5C –Final clinical aspect after 3 months; 5D – Final clinical aspect after 19 months.

In a later appointment, an acrylic resin shell (Duralay) was manufactured in the mouth over the intermediate abutment for later melting into a coping for the implant-supported crown. Periodontal surgery started after adjustments of the provisional crown. The operator, with previous experience in periodontal surgical techniques, selected an adaptation of the pinhole periodontal procedure [10] and of the technique proposed by Tarnow [11]. Initially, the patient was anesthetized with articaine 4% + epinephrine 1:100,000 (DFL®, Rio de Janeiro), and then two horizontal 2-mm incisions were made, separated by 3 mm of intact gingival tissue, using blade #15 in the alveolar mucosa, close to the top of the implant (Fig. 4A). A curved papilla elevator was inserted at the incision sites, dislodging the mucogingival flap. Flap elevation was guided by visualization of the shape and movements of the instrument through the mucosa and the gingival tissue, keeping them intact without fenestration. Detachment was then extended coronally and horizontally in order to elevate the papillae around the implant. The gingival implant in the region of the alveolar ridge was preserved without any rupture. The interproximal extension of the flap allowed free movement, permitting its coronal repositioning, extending it beyond the crown-implant interface. The distinctive feature of the pinhole procedure [10] was observed at the time of flap stabilization. In that technique [10], the gingival flap is stabilized using a malleable, bioresorbable membrane with no need of sutures. When compared with the technique proposed by Tarnow [11], which uses a semilunar incision, the current technique with two 2-mm incisions is aimed at reducing the surface area of the incision, allowing for larger vascularization of the flap at all times, which may improve healing of soft tissues later on. After detachment of gingival tissues, making them flabbier, the flap was stabilized using horizontal mattress suture (nylon 4.0) through the incision site with the knot tied in the palatal gingiva, thus preserving the gingival papillae (Fig. 4B). At the end of the procedure, the patient was informed about the postoperative protocol, which included specific care for the manipulated region, and about the use of medications (antibiotic and anti-inflammatory drugs). The gingival mucosa was assessed using a periodontal probe moved horizontally (Fig. 5A).

After 10 days, the stitches were removed, coping on the abutment was checked, and the colors for the ceramic composite were selected. In the subsequent appointment, the tooth was cemented with zinc phosphate (SS White) and the patient received hygiene instructions.

A graduated periodontal probe was used for patient evaluations. After 10 postoperative days, the gingival tissue showed satisfactory healing, with no signs of recession, infection, or inflammation, and no intercurrent events (Fig. 5B). After 3 months, there was remarkable improvement of gingival retraction, the peri-implant tissues were healthy, and gingival morphology was appropriate. In addition to gingival alignment with the adjacent teeth, the gingival papillae properly filled the interdental spaces (Fig. 5C). In the follow-up visit at 19 months, the peri-implant tissues were healthy without gingival retraction, and the morphology was preserved, with presence of keratinized gingival tissues along the anatomic shape of the prosthetic rehabilitation (Fig. 5D). At the end of treatment, the patient seemed satisfied with the esthetic outcome.

## 3 Discussion

Prosthetic planning prior to surgery is one of the key aspects of esthetic rehabilitations over anterior implants. When this planning is not appropriate, there could be intercurrent events that will probably affect the treatment outcomes.

After assessing the positioning of implants, it is important to select prosthetic components that are suitable to each clinical case, as they will guarantee clinical success for long time periods [12]. The selection of the prosthetic components in this case report was crucial for the final outcome. As pointed out in the literature, most dentists choose screw-retained prostheses because of their retrievability, allowing for repair and maintenance [8]. However, cement-retained prostheses have high success rates and quite favorable esthetics, but their maintenance is a bit more burdensome [13].

The patient described herein had a screw-retained prosthesis, but because of the surgical angulation of the implant, it was replaced with a cement-retained one. The outcome obtained in this case report is in line with that observed by other authors [13], with a good success rate for cement-retained implant-supported prostheses in esthetic zones.

Gingival retraction is an intercurrent event that may be present in regions subjected to dental implants, but this problem is worse in esthetic zones and it is not accepted by many patients. Regardless of whether the prosthesis is cement- or screw-retained, the characteristics of soft tissues around the implants are crucial for the desired esthetics and for the success of prosthetic rehabilitation [2].

Studies [14] have demonstrated gingival retraction after 5 years of follow-up in regions subjected to immediate implant placement and provisional crowns in esthetic zones. This finding is similar to the one reported in this clinical case, where the patient exhibited a 2-mm gingival retraction after the definitive crown placement, compared with the cervical region of tooth #21.

To improve gingival esthetics in these cases, subepithelial connective tissue grafting techniques are often necessary [15,16]. To avoid an extensive surgical procedure, two techniques were used: the pinhole surgical technique [10], in which the gingiva is repositioned without the use of grafts; and Tarnow’s technique [11], in which a semilunar incision is made in soft tissues. After the healing period, there was considerable improvement in the gingival margin (Fig. 5D).

It is also extremely important to carefully assess the forces applied by patients to obtain success in rehabilitation treatments [17]. Excessive forces are highly dangerous to prosthetic rehabilitations. Testing all movements during occlusal adjustment is a mandatory step, as excessive forces, especially shearing forces, overload the entire prosthetic set, which often does not bear the applied load, leading to intercurrent events such as screw fracture, porcelain fracture, screw loosening and, in extreme cases, implant fracture [1]. In this case report, the cracks in the porcelain may be associated with occlusal interferences. After some adjustments, the patient had no cracks or fractures in his new implant-supported porcelain crown.

The present clinical report is in line with the available literature [1], highlighting the importance of prosthetic planning prior to the surgical procedure. To solve esthetic and functional problems from previous treatments, innovations in surgical techniques are needed, as demonstrated in this report, simplifying the procedures and causing less morbidity to the patient.

## 4 Conclusion

Implant-supported prostheses in the anterior maxillary region have a high esthetic demand, and their placement is an extremely complex procedure. Factors such as prosthetic model, prosthetic components, presence or not of soft and hard tissues, masticatory forces applied by the patient, and alignment and positioning of the endosseous implant in the bone tissue are essential for achieving satisfactory results.

This clinical case underscored the importance of a multidisciplinary approach to obtain desired outcomes, such as the combination of prosthetic, periodontal, and surgical planning.

## Conflict of interest

The authors declare that they have no conflicts of interest relative to the publication of this manuscript.

## Funding

This study did not receive any specific grant from public, commercial, or nonprofit funding agencies.

## Ethical approval

The study is exempt from ethnical approval in our institution.

## Consent

The patient gave consent for the possible publication of his case report.

## Author contribution

Gustavo Vargas Da Silva Salomão: Performed the surgical procedure and drafted the manuscript.

Fernando Toledo dos Santos: Performed the prosthetic rehabilitation.

Sergio Allegrini Junior: Served as the advisor and revised the manuscript.

## Registration of research studies

There is no need, because this surgical technique used in this clinical case, have already been performed on patients and published, therefore, I believe it would not be necessary to have a new research register (UIN).

## Guarantor

Gustavo Vargas Da Silva Salomão.

Fernando Toledo Santos.

Sergio Allegrini Junior.

## Provenance and peer review

Not commissioned, externally peer reviewed.
